# Colorado State Veterinary Medical Association

**Published:** 1896-12

**Authors:** D. P. Frame

**Affiliations:** Secretary


Secretary.

### COLORADO STATE VETERINARY MEDICAL ASSOCIATION.

THE semi-annual meeting was called to order at 3 P.M., October 28th, at the office of the President, Dr. S. Bock, 1250 Glenarm Street, Denver, the President Dr. Sol. Bock in the chair. The following members answered to roll-call: Drs. S. Bock, F. W. Hunt, Charles G. Lamb, Charles Gresswell, W. A. Rushworth, E. Pouppirt, all of Denver; D. P. Frame, of Colorado Springs; and Dr. A. J. Savage, of Colorado Springs, visitor.

The principal object of calling a meeting at this time was to consider the matter of legislation in behalf of the profession in this State this winter. The old bill, which was introduced two years ago, was taken up and reconsidered, and after some few alterations in the phraseology, and one or two amendments were made to it, it was considered satisfactory. The principal amendment was that of Section 5, permitting non-graduates who have practised continually in the State for five or more years to register under the new law.

Moved by Dr. Gresswell that the regular meetings of this Association in the future be held on the Tuesday following the first Monday of January and July of each year; carried.

Dr. Frame proposed the name of Dr. A. J. Savage for membership, and the doctor was at once elected a member of this Association.

Drs. Gresswell and Rushworth were appointed to read a paper on "Glanders" at the meeting in January.

Dr. D. P. Frame was appointed to superintend the introduction of the proposed new bill in the Legislature this winter, and to represent the Association in the consideration of the same.

It is proposed to now hold regular semi-annual meetings of the Association, as we have been organized for over two years and have been making some effort to secure legislation in our behalf. We hope to succeed in our effort this winter.

The next meeting of the Association will be held in Denver, January 5, 1897.

D. P. Frame, M.D.C.,
Secretary.

### PENNSYLVANIA STATE BOARD OF VETERINARY MEDICAL EXAMINERS.

Secretary.

W. Horace Hoskins,

President.

### PERSONAL.

The many friends of Dr. Cecil French, one of our regular contributors, will be pained to learn that his recent marriage has been followed by the serious illness of his wife with typhoid fever, supposed to have been contracted at some one of the hotels where they had stopped on their wedding tour.

Veterinarian A. W. Bitting, of the Purdue Experiment Station, Lafayette, Ind., has just issued a bulletin relative to the prevalence of swine-plague, advising methods to prevent the great losses resulting therefrom.

Dr. T. J. Turner, recently at Kansas City, Mo., was transferred to Indianapolis to take charge of the inspection work of the Bureau of Animal Industry.

Dr. W. L. Zuill, of Philadelphia, has recently become the proprietor of two of Philadelphia's most fashionable and prosperous livery- and boarding-stables.

Dr. Charles H. Higgins, a recent graduate of McGill University, has located at Port Antonio, Jamaica, West Indies.

Dr. F. H. Osgood has been unanimously reëlected Chairman and Mr. Leander F. Herrick, of Worcester, Secretary of the Massachusetts Cattle Commission.

Dr. J. M. Parker qualified as a member of the Cattle Commission of Massachusetts on October 21, 1896.

The JOURNAL seems not to have been reliably informed of the salary of Dr. M. E. Knowles at the Marcus Daly Farm in Montana. In place of $4000, as stated, Dr. Knowles received $5500 per annum. The present salary is said to be fixed at $2000 per year.

Veterinarian G. H. Davison, of Milbrook, was recently appointed a member of the board of control of the New York State Experiment Station.

During November State Veterinarian Leonard Pearson, of Pennsylvania, addressed the Farmers' Institute, at Somerset, on the subject of "Hygiene as Applied to Farm Animals;" at Myersdale, on "Lameness of Horses;" and at Mt. Pleasant, on "Tuberculosis of Cattle."

Veterinarian Harry Emery, of Wilkinsburg, Pa., a graduate of the Ohio Veterinary College at Cincinnati, has opened a shoeing-forge in connection with his practice.

The reëlection of President John R. Hart and Secretary W. L. Rhoads as officers of the Keystone Veterinary Medical Association of Philadelphia and vicinity was a just recognition of their efficiency in conducting the affairs of the Association.

Professor John W. Adams recently addressed the Veterinary Medical Association of the Students of the Veterinary Department of the University of Pennsylvania on the subject of antitoxins and the care and methods of their preparation. Dr. Adams is officially connected with one of the largest laboratories for the production of these preparations.

The November meeting of the Keystone Veterinary Medical Association was one of exceptional interest. The address of Dr. Benjamin Lee, Secretary of the State Board of Health, was fraught with salient points of interest as to needed legislation on sanitary affairs. A large number of the students of the Veterinary Department of the University of Pennsylvania were present.

Dr. M. E. Knowles, of Butte, Montana, still lingers at Terre Haute, Ind., owing to the illness of his children.

Veterinarian James Marshall, of Philadelphia, was a time-keeper at the trial of speed of the famous pacer John R. Gentry, at Belmont track.

Professor Eggling, of Germany, sent here to make an examination of the mare Bethel, now so famous, has returned to Germany to make reports to the courts so that the guilt of Robert Kneebs may be finally decided.

Dr. Edward H. Flood, chief inspector of cattle for foreign shipment at the port of Philadelphia, under the Bureau of Animal Industry, has severed his connection therewith.

Dr. Alexander Glass, of Philadelphia, is said to be an applicant for the position of inspector under the Bureau of Animal Industry.

Governor Hastings, of Pennsylvania, has reappointed Dr. James W. Sallade, of Pottsville, a member of the State Board of Veterinary Medical Examiners. Dr. Sallade well merits this recognition, for he has been one of the most faithful members of the Board, and the Governor's action will meet the hearty approval of the profession and the other members of the Board.

Dr. William Dougherty, of Baltimore, has again been a sufferer from his old enemy, rheumatism, but we are glad to say he is getting better, and the wishes of his many friends are for a long period of freedom from this painful and distressing condition.

Dr. S. H. Ward, of Selkirk, Manitoba, has removed to St. Cloud, Minnesota, where he succeeds to the practice of Dr. W. H. Scruby. The latter has entered the Chicago Veterinary College preparatory for the degree of that institution.

Dr. Otto Noack, of Reading, addressed the Bucks County Agricultural Society meeting in November, referring especially to the importance and need of thorough measures in dealing with bovine tuberculosis.

Veterinarian E. C. Switzer, of Brimfield, Mass., is taking a course of instruction at the United States College of Veterinary Surgeons this winter.

Dr. R. D. Martin, of Bridgeport, Conn., has been elected one of the Board of Governors of the Bridgeport Driving-club.

Dr. N. P. Hinkley, of Buffalo, has again been forced to relinquish much of his professional work owing to impaired health.

President Hoskins, of the Pennsylvania State Board of Veterinary Medical Examiners, will address the Students' Association of the Veterinary Department of the University of Pennsylvania, in December, on the "Future Field of Work of the Veterinarian."

Dr. A. O. Cawley, of Milton, Pa., was one of the judges of horses at the Northumberland County Agricultural Show in October.

Corresponding Secretary Noack, of the Schuylkill Valley Veterinary Medical Association, was a welcome and interesting visitor to the November meeting of the Keystone Veterinary Medical Association.

Dr. O. G. Atherton, of Kansas City, Kansas, was recently appointed Assistant Inspector, and assigned for duty at Chicago.

---

MESSRS. P. BLAKISTON, SON & CO., 1012 Walnut Street, Philadelphia, have issued their annual *Physician's Visiting List* for 1897. The improvements in this very useful book, which added so much to its increased worth in 1896, have been still further added to for 1897, making it of the most complete character. This book is quite well adapted for the use of veterinarians, and its concise method of keeping one's accounts, whereby a ready statement is to be provided, renders it of additional value.

---

### EVERY-DAY HAPPENINGS.

A school of farriery will be organized at Buffalo this winter, and among the lecturers already engaged we learn of the selection of Dr. Nelson P. Hinkley.

There was only one candidate for exa mination for meat inspector at the examination held in Kansas City, Kansas, on Monday, October 26th. It is thought that the large percentage of failures to successfully cope with former examinations was the reason for such a small number of applicants, as the desire to secure these positions by Western veterinarians has been very strong, owing to the fact that practice in many of the Western States has been so unprofitable.

Dr. Newton S. Bryant, of Kansas City, a veterinary surgeon, who has been in New York for the past two months, was arrested at the horse-show Tuesday evening, November 10th, on a warrant charging him with practicing in New York State without being properly registered. The warrant was issued at the instance of Dr. Rush S. Huidekoper, member of the New York State Board of Veterinary Medical Examiners. The case has been postponed for a time.

At the Royal Veterinary College the inaugural address was delivered by Professor Macqueen, F.R.C.V.S. He said that twenty-one years ago the education of veterinary students was a simple affair. Candidates for admission were subjected to an elementary examination conducted voluntarily by the colleges. The professional studies occupied two sessions of seven months each. Now the preliminary examination is the same as that required from students of human medicine. The course of professional study has been extended to four years. To qualify for the diploma (M.R.C.V.S.) a student must attend 700 lectures, 200 demonstrations, and more than that number of clinical and tutorial classes. Further, he must attend the examination of 1800 or more animals and watch the progress of the patients. He must take a share of the practice at the Free Clinique, where 5000 animals—horses and dogs—are treated every year. Lastly, he must take an active interest in the examination as to soundness of ten or eleven hundred horses of all classes.—*London Times*, October 9, 1896.

### GLEANINGS.

Iodide of potassium and salicylate of soda in half-drachm doses each, combined with one drachm of wine of colchicum, with sufficient water to make one ounce, given four times daily in conjunction with a cooling lotion and bandages, eradicated quickly and effectively an acute attack of rheumatism involving the front limbs from the knees down, at the Philadelphia Veterinary Sanitarium.

"Probasco," a noted running horse, after an injection of cocaine in an unsound limb, entered upon a mad run upon the race-track at Cincinnati, Ohio, and fractured the injected leg, necessitating his destruction. Hence the posting of Rule 187, absolutely prohibiting the giving of drugs to a horse just before a race in which it is to take a part, and its strict enforcement; and no excuse that they were given for medicinal purposes will be accepted. Horses needing such remedies must not be raced.

The whole world is awakening to the real importance of bovine tuberculosis, and every advanced community appreciates the necessity of active warfare against this source of tuberculosis, and its stamping out among our dairy-herds will be accomplished at no distant day by legislative enactments in every country and State. No matter what difference of opinion may exist as to whence it has come into our herds, it is our duty to stamp it out, and, if not done through a united profession and a consenting agricultural people, there will be to the latter the greatest losses ever experienced in the decreased consumption of their products and the search for substitutes, which has already begun, and when perfected will mean a permanent loss. The medical press is teeming with reports, criticisms, and editorials on the all-important need of a pure milk-supply, and, alive to the dangers of an unwholesome product, aside from the fact of the existence of tuberculosis, demands its production, sale, and care under greater sanitary safeguards; and it is well, it is right and proper; and an increased value in the open market will more than compensate the producers and advance the interests of all who are engaged in the production and sale of animal food-products.

It is interesting to note at home and abroad the deep concern of the profession in the transmission of hereditary predisposition to certain unsoundnesses and malformations in the equine species. In these days, when these blemished and defective animals are so great a drug in the market, it is well to agitate greater safeguards against their perpetuation, and the incident, direct and indirect, loss they cost the raiser, the dealer, and the veterinarian. It is quite time that State laws should be adopted having jurisdiction over stallions and bulls offered for service, that an innocent and confiding public may be protected from certain dangers and losses in this direction, the perpetuation of which only means greater hardships to all engaged in the live-stock industry.

